# Werner Nachtigall (1934–2024)

**DOI:** 10.1007/s00359-024-01726-1

**Published:** 2024-11-26

**Authors:** Reinhard Blickhan

**Affiliations:** Science of Motion, Friedrich-Schiller-University, 07749 Jena, Germany

**Keywords:** Obituary, Biophysics, Bionics, Flight, Insects, Birds

## Abstract

On September 5th, 2024, Werner Nachtigall (born July 6, 1934) passed away. Nachtigall is a pioneer in biomechanics, a founder of biomimetics (bionics), and a relentless advocate for his field in Germany. He conducted broad-ranging and innovative work on biomechanics in insects, birds, and fishes. He developed elaborate technical methods, such as sensitive scales, wind-tunnels, and high-speed photography. The research he performed at the Ludwig-Maximilians-University in Munich (dissertation and habilitation) and especially as a full professor at the Saarland-University in Saarbrücken focused on the biophysics of swimming in insects, and flight in both insects and birds. He set new standards for kinematic, aerodynamic, energetic, and cybernetic investigations. With his team, he continued to expand his biological and technical interests, ranging from the biomechanics of fish locomotion to the mechanics of biological light weight structures. With Werner Nachtigall we lost a talented scientist, a dedicated teacher, an enthusiastic naturalist, and a highly productive author.

## Biography

**Fig. 1:**
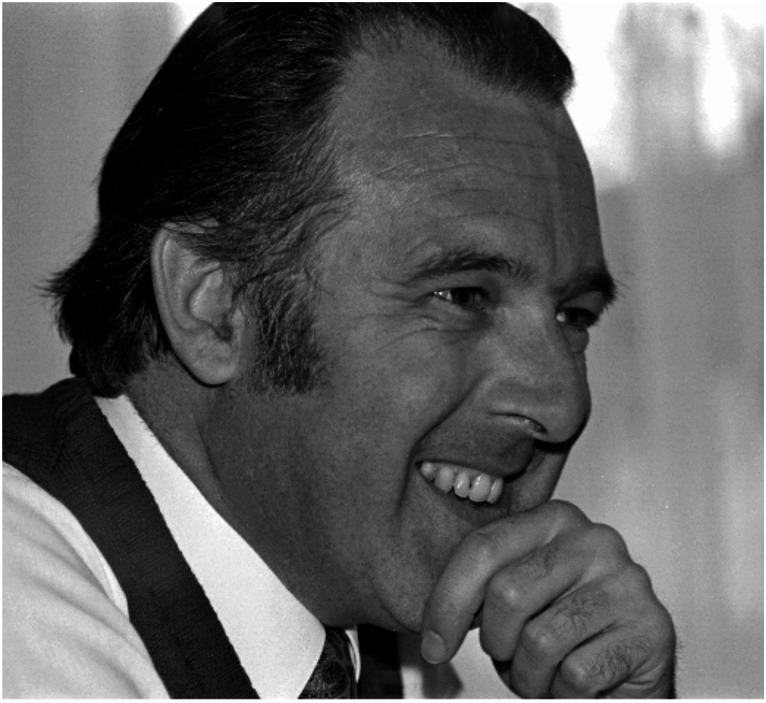
Werner Nachtigall 1976. (Photograph: Helmut Nachtigall)

Remaining rather healthy and alert with only moderate limitations in the last three years, Werner Nachtigall (Fig. 1) suddenly passed away on September 5th, 2024. He survived his wife Martha (passed away on December 29, 2021), and he leaves behind his daughter Irene and his brother Helmut. He was laid to rest near his home at the Scheidter Berg, a small village close to Saarbrücken, Germany.

Werner (Othmar Wenzel Albert) Nachtigall was born in Saaz, Bohemia, (now Žatec, Czechia). His father was a clerk in a company processing hops. At the end of World War II, eleven-year-old Werner, being a German inhabitant, was imprisoned together with his mother Lilly and his brother, and finally deported to Dasing/Augsburg, Bavaria (Nachtigall 1987). Here, he finished (in 1954) nine-year high school (*Gymnasium bei St. Anna* and *Realgymnasium*). At the Ludwig-Maximilians-University, Munich, he started as a working student. Later, he was supported by the German Academic Scholarship Foundation. He chose to study biology (his teachers included Karl von Frisch and Karl Mägdefrau), as well as chemistry and geography. To broaden his scientific knowledge, he also attended courses in zoology, physics with Walther Gerlach, and human physiology with Richard Wagner. In parallel, he studied fluid mechanics and aeronautical engineering with Erich Truckenbrodt at the Technical University, Munich. During his studies, he met his life-long partner, Martha John (John 1958), who was his tutor in a chemistry course and a specialist in insect hormones. They married in 1960. Instead of becoming a high school teacher, Nachtigall chose to focus on science and finished his studies in 1959 with a PhD (Dr. rer. nat., *summa cum laude*) under the supervision of Werner Jacobs. His dissertation on the swimming behavior of whirligig beetles (Nachtigall 1959) paved the way for his future investigations in biophysics. At the Institute for Biology of Radiation at the University in Munich, affiliated with the *Gesellschaft für Strahlenforschung Neuherberg* (currently: Helmholtz Munich), he analyzed the influence of x-ray radiation on the processing of visual information in insects (Nachtigall 1965), instead of simply focusing on damage caused by radiation. After two years, he decided to follow up his earlier line of research related to fluid mechanics and to return to the Institute of Zoology in Munich, where he worked under the supervision of Hansjochem Autrum. There, at the beginning with a fellowship from the German Science Foundation (1961), he deepened his studies on the locomotion of semiaquatic insects. Later, as an assistant professor, Nachtigall established a research team that included, among others, Wolfram Zarnack and Dietrich Bilo, and he extended his studies to bird flight. The subject of his habilitation (1966) was still insect flight. Meanwhile, in 1964, in her hometown (Überlingen), his wife gave birth to their daughter Lilly and then ceased her academic career. As a research fellow and assistant professor in Donald Wilson’s laboratory at the University of California at Berkeley (1967), Nachtigall augmented ongoing studies on the motor control of insect flight with his expertise in biophysics. With declining opportunities for full professorships in the U.S.A., he returned to the Institute of Zoology in Munich as a lecturer. In 1969, the Saarland-University, Saarbrücken, offered him a position as full professor and director of the Institute of Zoology where his predecessor was Gustaf de Lattin. There, he followed up his pioneering line of research with his talented team, reinforced by new members that included Bernhard Möhl and Klaus Pfau. He declined an offer from the Ludwig-Maximilians-University Munich in 1976. From 1990 to 1992, he was dean of the faculty of Mathematics and Natural Sciences at the Universität des Saarlandes. He retired in 2002. In private, Nachtigall liked to play classical music on the piano and the violin. Music and the history of old churches, combined with his wife’s accomplishments in ceramics, demonstrated his wide interests beyond science throughout his life.

## The scientist

As a child in Saaz, Nachtigall was fascinated by the shiny objects that often appeared in formation flight on the sky, not knowing about their deadly load (Nachtigall 1987). Curious about flight, he went on to spend most of his scientific carrier investigating the dynamics and energetics of animal flight, where flexible appendages and body parts provide lift and propulsion. For his investigations, he applied and built instruments for kinematic recordings, scales to measure fluid dynamic forces, as well as cages, basins, flow channels, and tunnels to provide controlled conditions for the study of flight and swimming. Nachtigall used comparative morphological and biophysical studies to establish basic principles. He became a highly productive scientific author (Zupanc et al. 2024). Here are selected highlights of some of his research.

In his doctoral thesis, he built a stroboscope combined with a commercial 16 mm camera to track markers attached to the appendages of diving beetles (Coleoptera, Dytiscidae) and a spring scale to measure body drag (Nachtigall 1960). Later, supported by the German Science Foundation, he constructed a 16 mm high speed camera enabling 800 frames/s which, in combination with a strobe light, enabled a stroboscobic micrograph within each high-speed frame. His observations stressed the efficiency of the leg morphology used in swimming. As a closing statement, Nachtigall (1961) writes: “It is the best-known thrust apparatus in the animal kingdom making use of the resistance principle”. Measurements on models of the beetle’s body in a water tunnel, a wind tunnel, and a towing tank yielded information on drag optimization and stability of the dytiscid body (Nachtigall and Bilo 1965). He employed these developed techniques to illuminate the kinematics and locomotion patterns of the larvae of different flies (Dipterae) and diving beetles (Dytiscidae).

A major advancement in the investigation of flight was the use of the tethered fly. A fly was fixed on a mechanical 3D-scale to control flight in front of a wind tunnel, allowing unpreceded recordings of wing movements at different wind speeds, thus significantly enhancing our understanding of insect flight (Nachtigall 1966). Later these findings were complemented by the consideration of unsteady effects (Nachtigall 1979a). In his investigation on the flight dynamics of honey bees, Nachtigall and his coworker Hanauer-Thieser could show that body and legs contribute considerably to lift (Nachtigall and Hanauer-Thieser 1992). Again, newly designed pieces of equipment, such as a round-about, a closed ring-chamber for insect flight, and a closed wind-tunnel with a low volume, allowed detailed investigations of the energetics of bee flight (Rothe and Nachtigall 1989; Nachtigall et al. 1995). During his visit to Wilson’s laboratory, Nachtigall investigated how different muscles contribute to the control of the wing kinematics (Nachtigall and Wilson 1967; Nachtigall 1968a). Later, this line of research was pursued by his team member Bernhard Möhl.

In parallel, Nachtigall’s established methodical advances were adapted and enhanced to illuminate the mechanics and energetics of bird flight. Early studies focused on aerodynamic profiles during gliding flight (Nachtigall and Wieser 1966). Nachtigall (1998b) details a comprehensive summary of further developments and new experimental stations, such as large-scale wind tunnels in the laboratories in Saarbrücken equipped with instruments for stereo-photogrammetry, and respiratory measurements. Among others, these studies delivered information about thermoregulation and water homeostasis in pigeons (Biesel and Nachtigall 1987). Long-distance flight seemed not to be limited by fuel but by the fact that evaporative water loss exceeded metabolic water production. The cybernetics of bird flight became a main subject of his early and long-lasting team member Dietrich Bilo.

Nachtigall’s expertise helped to interpret the function of the lobed fins of the coelacanth (Fricke et al. 1987). To further advance our understanding of fish locomotion, he designed a facility that included tanks and flow tunnels to investigate the biophysics of undulatory swimming in fish (Blickhan et al. 1992; Kesel et al. 1989). These studies notably characterized the efficiency of undulatory propulsion based on kinematics and the flow in the wake, as well as muscle recruitment.

Within the special research field of the German Science Foundation “Constructions in Nature” (SFB 230 *Natürliche Konstruktionen*) funded from 1984 to 1995, participants worked to achieve a general understanding of natural and technical constructions. As an expert in biomechanics and bionics, Nachtigall made significant contributions to this research program. His projects included research on the mechanical stability of grass in the wind tunnel (Nachtigall et al. 1986). His interest on biological light weight structures is also indicated by investigations of the mechanical properties of insect wings (e.g. Kesel et al. 1998).

Always with an eye on science, Nachtigall was also a naturalist. To go for a walk with him meant to be confronted step-by-step with the Latin names for almost everything seen. In numerous small projects, he documented behaviors in the wild of flight in seeds, insects, and birds. Using his private equipment, he evaluated the data and published the results in a series of twenty-one articles for the interested entomologist (e.g., Nachtigall 2018).

## The teacher

Nachtigall was an excellent teacher. To his team he provided opportunities and guidance, while encouraging them to follow their own directions. In his lectures he was always prepared, developed his thoughts step-by-step, and provided clear, thorough, and up-to-date material for his students. During his lecture on statistics, he developed information formula by formula, line by line. His basic and advanced courses on physiology covered a wide range of preparations and experiments on animal physiology. Lectures on physiology included human physiology and cybernetics. The introductory course on biomechanics covered basic concepts in mathematics as well as advanced methods in fluid mechanics. To support education, Nachtigall published material as textbooks (statistics: Kesel et al. 1999; physiology: Nachtigall 1981a, b; biomechanics: Nachtigall 2001a; Nachtigall 2001and Nachtigall 2001a chapter in a pioneering textbook on biophysics: Mannherz et al. 1977). Nachtigall had a passion for morphological details, and he shared his experience on microscopy in a series of articles in the journal Mikrokosmos to a wide audience (e.g., Nachtigall 2001b), culminating again in a textbook (Nachtigall et al. 2020). He offered seminars on the consequences of the human impact on the environment at a time when discussions about ecological consequences of human actions on ecology had just begun to surface. Supported by his academic staff, he organized field trips within Germany and to nearby France, and marine biology excursions to coastal sites in France, Croatia, and Sicily.

## The communicator

**Fig. 2:**
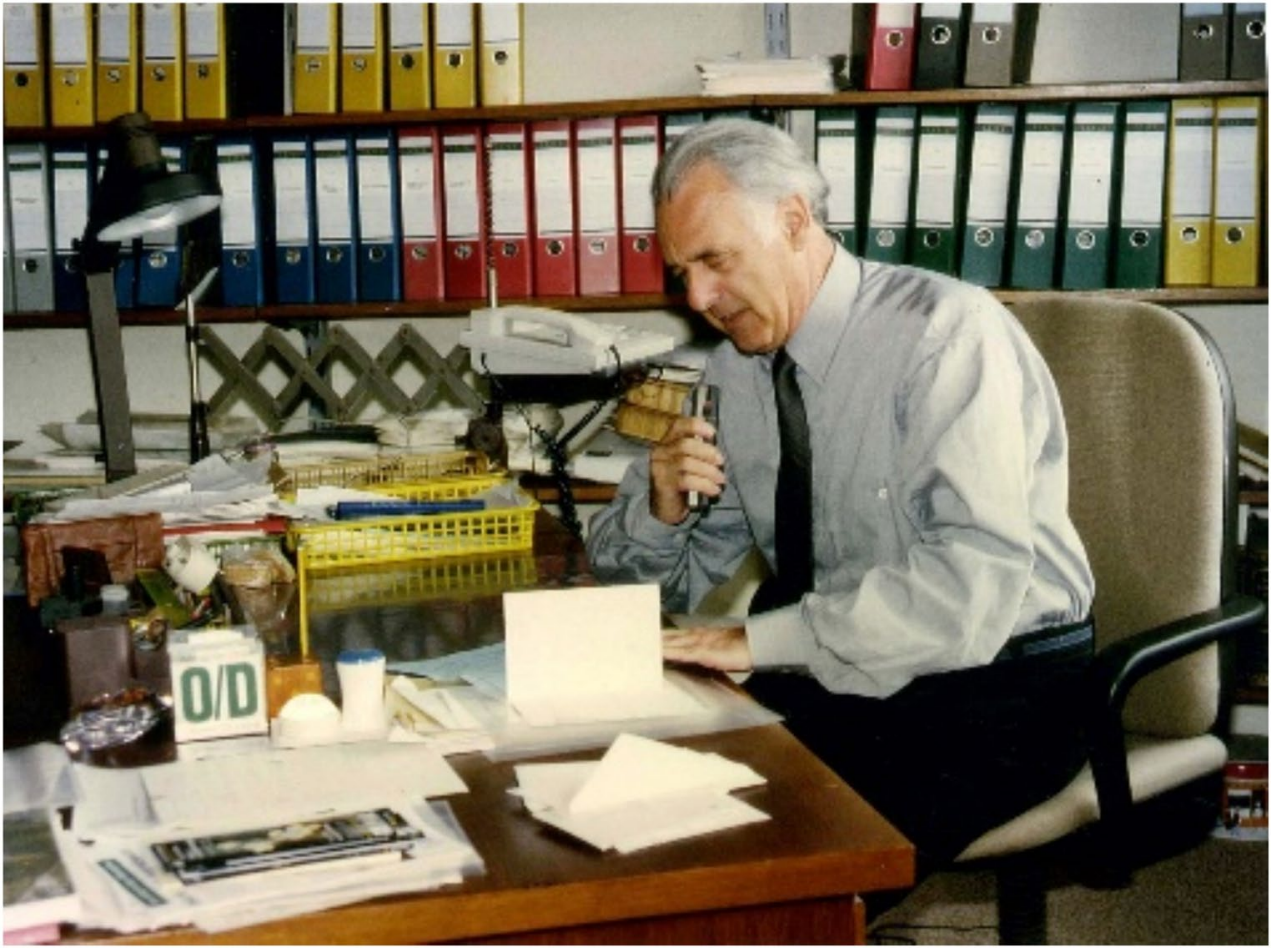
Werner Nachtigall dictating a text in his office at the Institute of Zoology in Saarbrücken (Photograph: Helmut Nachtigall)

Nachtigall reached a wide audience by presenting his observations and ideas to the public. In an early book, he combined biophysical information with exceptional photography (Nachtigall 1968b). He fluently dictated whole book chapters into a voice recorder (Fig. 2; “*Ich schreibe eben gerne* - I just like writing”, Nachtigall 2023). Equipped with a collection of graphic and photographic material, his team supported his writing activities. Nachtigall had an interest in communicating science to the general public and he put his research into a wider context (Nachtigall 1974, 1977, 1979b, 1983, 1985), even considering ecology and architecture (Nachtigall and Pohl 2013). He published more than one hundred books, including textbooks, books for the general public, and scientific books. Some of them appeared in multiple editions (Nachtigall 2023).

## The pioneer

Most prominently, Nachtigall was an expert in technical biology and a pioneer in bionics. In his work on the biophysics of locomotion in fluids he applied concepts from engineering to biological objects (biotechnics). Nevertheless, from his early days onward, he was also convinced that engineers could learn from nature by unraveling principles of construction and function of biological objects (bionics or biomimetics). He developed concepts which can be considered the foundation for emerging scientific fields (e.g., Nachtigall 1982, 1994, 1997a, b, 1998a; Nachtigall and Blüchel 2003). He rarely attended scientific conferences, but he communicated bionic advances in numerous presentations. In 1990, he founded the Society for Technical Biology and Bionics, Bremen, which still holds annual meetings under the leadership of his former student Antonia Kesel, who founded the Biomimetics Innovation Centre in Bremen in 2005. Furthermore, in 2001, Nachtigall was co-founder of a network (*Bionik-Kompetenznetzwerk*, BIOKON, Berlin) connecting entrepreneurs, engineers, and life scientists to accelerate transfer of scientific knowledge into engineering and commercial products. His efforts to establish the field of bionics not only succeeded, but many avenues continue to grow up to today.

His scientific accomplishments, his skills in communicating science, as well as his pioneering activities were recognized by a number of prestigious awards, including the Fabricius-Medal of the German Society for General and Applied Entomology for his biophysical investigations on insect flight (1970); the Karl-Ritter-von Frisch Medal of the German Zoological Society for his investigations on the physiology of animal locomotion, especially on the biophysics of flying and swimming, but also for his remarkable ability to communicate scientific results to a wider audience (1982); the constructional award of the Fritz-Bender-Foundation for the development of a ventilation system based on termite mounds (together with Georg Rummel, 1996); the International Rheinland-Award for protection of the environment by the Technical Control Board Berlin-Brandenburg-Rheinland (2002); and the Treviranus Medal of the Verband Deutscher Biologen, an umbrella organisation for life sciences in Germany for his pioneering work in bionics and for communicating related science to the general public (2004). He was a member of the Akademie der Wissenschaften und der Literatur Mainz (since 1979) and a member of the Sudetendeutsche Akademie der Wissenschaften und Künste, Munich (since 1987).

## Data Availability

No datasets were generated or analysed during the current study.

## References

[CR1] Biesel W, Nachtigall W (1987) Pigeon flight in a wind tunnel. J Comp Physiol B 157(1):117–128. 10.1007/BF00702736

[CR2] Blickhan R, Krick C, Zehren D, Nachtigall W, Breithaupt T (1992) Generation of a vortex chain in the wake of a subundulatory swimmer. Naturwissenschaften 79:220–221. 10.1007/BF01227131

[CR3] Fricke H, Reinicke O, Hofer H, Nachtigall W (1987) Locomotion of the coelacanth *Latimeria chalumnae* in its natural environment. Nature 329(6137):331–333. 10.1038/329331a0

[CR4] John M (1958) Über den Gesamtkohlenhydrat- Und Glykogengehalt Der Bienen (*Apis mellifica*). Z Vergl Physiol 41(2):204–220. 10.1007/BF00345586

[CR5] Kesel A, Blickhan R, Nachtigall W (1989) Ablation of the lateral line organ. Does it affect steady swimming? In: Elsner N, Singer W (eds) Dynamics and plasticity in neural systems. Georg Thieme, Stuttgart, p 256

[CR7] Kesel AB, Philippi U, Nachtigall W (1998) Biomechanical aspects of the insect wing: an analysis using the finite element method. Comp Biol Med 28(4):423–437. 10.1016/S0010-4825(98)00018-3 10.1016/s0010-4825(98)00018-3 9805202

[CR6] Kesel AB, Junge MM, Nachtigall W (1999) Einführung in die angewandte Statistik für Biowissenschaftler. Birkhäuser Basel, Basel. 10.1007/978-3-0348-8702-1

[CR8] Mannherz HG, Holmes KC, Nachtigall W, Bauer RD, Pasch T, Wetterer E, Ziegler H, Zwicker E, Neuweiler G (1977) Biomechanik. In: Hoppe W, Lohmann W, Markl H, Ziegler H (eds) Biophysik: Ein Lehrbuch. Springer Berlin Heidelberg, Berlin, Heidelberg, pp 502–600. 10.1007/978-3-642-96298-1_14

[CR10] Nachtigall W (1959) Über Kinematik, Dynamik und Energetik des Schwimmens einheimischer Dytisciden: Zugleich ein Beitrag zur Anwendung von Kurzzeitphotographie und Hochfrequenzkinematographie auf biologische Probleme. Dissertation, Würzburg

[CR11] Nachtigall W (1960) Über Kinematik, Dynamik Und Energetik Des Schwimmens Einheimischer Dytisciden. Z Vergl Physiol 43(1):48–118. 10.1007/BF00351202

[CR12] Nachtigall W (1961) Funktionelle Morphologie, Kinematik Und Hydromechanik Des Ruderapparates Von *Gyrinus*. Z Vergl Physiol 45(2):193–226. 10.1007/BF00297764

[CR13] Nachtigall W (1965) Untersuchungen am Elektroretinogramm über die Wirkung Ionisierender Strahlen auf das Komplexauge Von Insekten. Biophysik 2:145–165. 10.1007/bf01185763 5855263 10.1007/BF01185763

[CR14] Nachtigall W (1966) Die Kinematik Der Schlagflügelbewegungen Von Dipteren Methodische und analytische Grundlagen Zur Biophysik Des Insektenflugs. Z Vergl Physiol 52(2):155–211. 10.1007/BF00343160

[CR15] Nachtigall W (1968a) Elektrophysiologische Und Kinematische Untersuchungen über Start Und Stop Des Flugmotors Von Fliegen. J Comp Physiol A 61(1):1–20. 10.1007/BF00339142

[CR16] Nachtigall W (1968b) Gläserne Schwingen. Heinz Moos, München

[CR17] Nachtigall W (1974) Phantasie der Schöpfung. Hoffmann und Campe, Hamburg. 10.1002/biuz.19750050212

[CR18] Nachtigall W (1977) Funktionen Des Lebens: Physiologie Und Bioenergetik Von Mensch, Tier Und Pflanze. Hoffmann und Campe, Hamburg

[CR19] Nachtigall W (1979a) Rasche Richtungsänderungen und Torsionen Schwingender Fliegenflügel Und Hypothesen über zugeordnete instationäre Strömlingseffekte. J Comp Physiol 133(4):351–355. 10.1007/BF00661137

[CR20] Nachtigall W (1979b) Unbekannte Umwelt: die Faszination Der Lebendigen Natur. Hoffmann und Campe, München

[CR21] Nachtigall W (1981a) Zoologischer Grundkurs. Wiley-Verlag Chemie, Weinheim

[CR22] Nachtigall W (1981b) Zoophysiologischer Grundkurs. Wiley-Verlag Chemie, Weinheim

[CR23] Nachtigall W (1982) Biotechnik Und Bionik-Fachübergreifende Disziplinen Der Naturwissenschaft. Franz Steiner, Wiesbaden

[CR24] Nachtigall W (1983) Biostrategie: eine Überlebenschance für unsere zivilisation. Hoffmann und Campe, München

[CR25] Nachtigall W (1985) Warum die Vögel fliegen. Rasch und Röhrig, Hamburg-Zürich

[CR9] Nachtigall L (1987) Als die Zeit Stehen Blieb: Eine Sudetendeutsche Mutter Erlebt Internierung, Vertreibung Und Neuanfang. Heimatbrief Saazerland. Buchdruckerei Schöffl, Forchheim

[CR26] Nachtigall W (1994) Aspekte Der Struktur- Und Konstruktionsbionik. In: Nachtigall W, Schönbeck C (eds) Technik Und Natur. Springer Berlin Heidelberg, Berlin, Heidelberg, pp 205–226. 10.1007/978-3-662-01104-1_9

[CR27] Nachtigall W (1997a) Vorbild Natur: Bionik-Design für funktionelles Gestalten. Springer, Berlin, Heidelberg. 10.1007/978-3-642-60866-7

[CR28] Nachtigall W (1997b) Zehn Grundprinzipien natürlicher Konstruktionen – 10 gebote bionischen designs. In: Nachtigall W (ed) Vorbild Natur: Bionik-Design für funktionelles Gestalten. Springer Berlin Heidelberg, Berlin, Heidelberg, pp 21–34. 10.1007/978-3-642-60866-7_4

[CR29] Nachtigall W (1998a) Bionik — was ist das? In: Nachtigall W (ed) Bionik: Grundlagen Und Beispiele für ingenieure und Naturwissenschaftler. Springer Berlin Heidelberg, Berlin, Heidelberg, pp 3–15. 10.1007/978-3-662-06114-5_1

[CR30] Nachtigall W (1998b) Starlings and starling models in wind tunnels. J Avian Biol 29(4):487–484. 10.2307/3677167

[CR31] Nachtigall W (2001a) Biomechanik Grundlagen, Beispiele, Übungen. Vieweg und Teubner, Wiesbaden. 10.1007/978-3-663-01611-3

[CR32] Nachtigall W (2001b) Über mikroskopisches Zeichnen. Mikrokosmos 90(4):248–250

[CR33] Nachtigall W (2018) Insect flight patterns in the natural environment-a retrospective. Entomol Gener 37(3–4):243–259. 10.1127/entomologia/2018/0649

[CR34] Nachtigall W (2023) Werner’s Berufsleben: München, Berkeley, Saarbrücken. Amazon. Independently published, Wroclaw

[CR35] Nachtigall W, Bilo D (1965) Die Strömungsmechanik Des Dytiscus-Rumpfes. Z Vergl Physiol 50(4):371–401. 10.1007/BF00339425

[CR36] Nachtigall W, Blüchel K (2003) Das große Buch Der Bionik: neue Technologien Nach dem Vorbild Der Natur. Deutsche Verlags Anstalt, München

[CR37] Nachtigall W, Hanauer-Thieser U (1992) Flight of the honeybee. J Comp Physiol B 162(3):267–277. 10.1007/BF00357534 1613166 10.1007/BF00357534

[CR40] Nachtigall W, Pohl G (2013) Bau-Bionik: Natur - Analogien - Technik. Springer Vieweg, Berlin. 10.1007/978-3-540-88995-3

[CR41] Nachtigall W, Wieser J (1966) Profilmessungen am Taubenflügel. Z Vergl Physiol 52(4):333–346. 10.1007/BF00302288

[CR42] Nachtigall W, Wilson DM (1967) Neuro-muscular control of dipteran flight. J Exp Biol 47(1):77–97. 10.1242/jeb.47.1.77 6058982 10.1242/jeb.47.1.77

[CR43] Nachtigall W, Wisser A, Wisser C (1986) Pflanzenbiomechanik (Schwerpunkt Gräser). Konzepte SFB 230 230(24):12–22

[CR38] Nachtigall W, Hanauer-Thieser U, Mörz M (1995) Flight of the honey bee VII: metabolic power versus flight speed relation. J Comp Physiol B 165(6):484–489. 10.1007/BF00261303

[CR39] Nachtigall W, Piper J, Fox F (2020) Faszination Mikroskopie: Band 1 Grundlagen-Techniken-Anwendungen. Hachinger Verlagsgesellschaft, Oberhaching-Munich

[CR44] Rothe U, Nachtigall W (1989) Flight of the honey bee. J Comp Physiol B 158(6):739–749. 10.1007/BF00693012

[CR45] Wikipedia https://de.wikipedia.org/wiki/Werner_Nachtigall. Accessed 23.09.2024

[CR46] Zupanc GKH, Homberg U, Helfrich-Förster C, Warrant EJ, Simmons AM (2024) One hundred years of excellence: the top one hundred authors of the Journal of comparative physiology A. J Comp Physiol A 210(2):109–144. 10.1007/s00359-024-01699-1 10.1007/s00359-024-01699-1 PMC10995051 38551673

